# Significance of outer retinal undulation on preoperative optical coherence tomography in rhegmatogenous retinal detachment

**DOI:** 10.1038/s41598-020-72907-6

**Published:** 2020-09-25

**Authors:** Young Do Yeo, Yu Cheol Kim

**Affiliations:** Department of Ophthalmology, Keimyung University School of Medicine, 1095 Dalgubeol-daero, Dalseo-gu, Daegu, 42601 Republic of Korea

**Keywords:** Diseases, Risk factors, Signs and symptoms

## Abstract

Rhegmatogenous retinal detachment (RRD) is a vision-threatening pathology. Optical coherence tomography (OCT) is useful for evaluating retinal damage and visual prognosis in patients with RRD. Outer retinal undulation (ORU) is often observed on preoperative OCT in RRD. Therefore, we evaluated the correlation between ORU seen on preoperative OCT and pre/post-operative factors in RRD. Patients with RRD (114 eyes) underwent reattachment surgery and ≥ 6 months of follow-up. According to the condition of the macula on preoperative OCT, cases were divided into macula-on RRD (65 eyes) or macula-off RRD (49 eyes). Patients were classified into acute (< 10 days), subacute (10–30 days), and chronic (> 30 days) symptom duration groups. Clinical findings, histories, and relationships with OCT findings, including ORU, were analyzed. Subacute symptom duration was significantly associated with ORU on preoperative OCT (*p* = 0.001) and had a higher prevalence of ORU (73.7%) than did acute (OR = 4.48) or chronic (OR = 7.467) durations. Ellipsoid zone (EZ) disruption was significantly associated with poorer best-corrected visual acuity (BCVA) than normal EZ integrity at 6 months postoperatively (*p* = 0.012). ORU on preoperative OCT suggests a 10–30 days morbidity duration in RRD. EZ integrity is useful for predicting postoperative BCVA in macula-off RRD.

## Introduction

Rhegmatogenous retinal detachment (RRD) is a vision-threatening pathology in which the degree of retinal damage is related to age, duration of morbidity, and the size of the affected RRD area^1^. Several studies have suggested that morbidity duration is a negative visual prognostic factor in macula-off RRD^2–7^. However, the reported retinal detachment period, which is based on subjective symptoms, may not be accurate.

Currently, optical coherence tomography (OCT) is used to evaluate retinal damage and visual prognosis in patients with RRD^8–11^. Lecleire-Collet et al. reported that both the detachment height at the fovea and the distance from the foveal center to the nearest undetached retina on preoperative OCT were associated with visual prognosis^8^. Park et al. found that six factors (ellipsoid zone integrity, the Henle’s fiber layer and the outer nuclear layer/photoreceptor layer ratio, the photoreceptor outer segment length, the photoreceptor inner segment length/photoreceptor outer segment length ratio, the ratio of photoreceptor layer thickness between the retinal detachment eye and fellow eye, and the photoreceptor outer segment length ratio between the retinal detachment eye and fellow eye) on preoperative OCT were prognostic factors for functional outcome in macula-off RRD^11^.

Outer retinal undulation (ORU) is often observed on preoperative OCT in RRD. Hagimura at el reported that 8/25 cases (32%) had ORU in macula-off RRD; however, they could not find a correlation between ORU and postoperative best-corrected visual acuity (BCVA)^12^. Lee et al. reported that 40% of macula-off RRD had ORU^13^. However, research on the association between ORU and pre/post-operative factors in RRD, including symptom duration, remains insufficient. The authors therefore evaluated the correlation between preoperative OCT findings, including ORU, and pre/post-operative factors, such as visual acuity and symptom duration, in RRD.

## Methods

**Figure 1:**
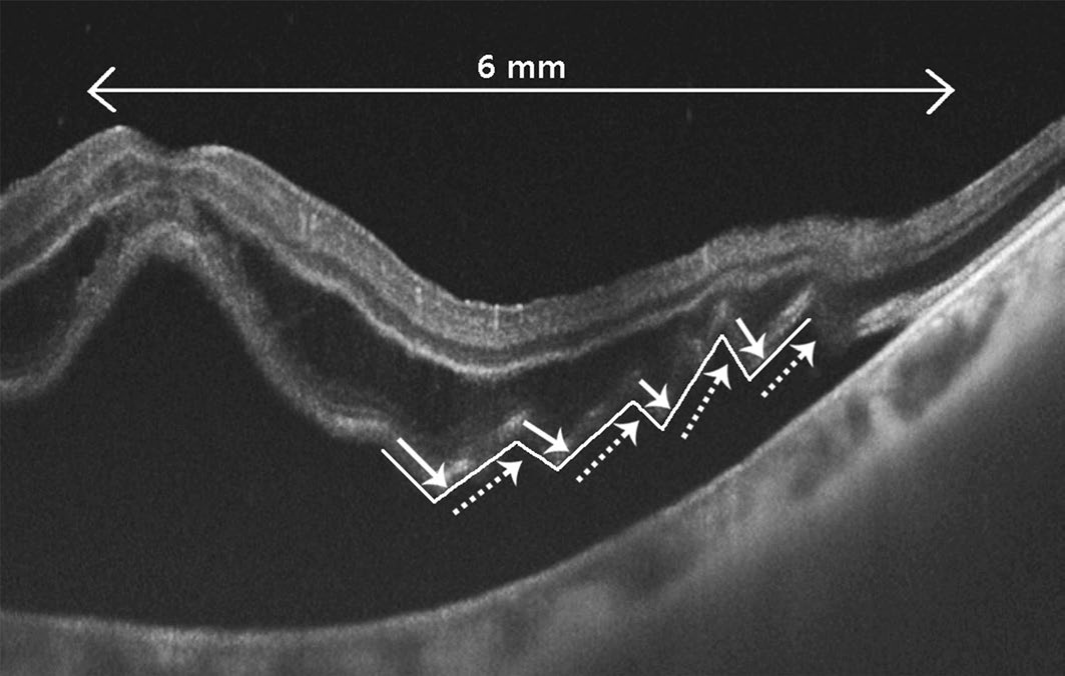
Definition of outer retinal undulation (ORU) on preoperative optical coherence tomography; lined arrow: negative directed undulation, dotted arrow: positive directed undulation. The presence of ORU is defined as > 3 combinations of negative and positive undulations (zig zag line).

We retrospectively reviewed the electronic medical records of 114 patients (114 eyes) who underwent surgical treatment for RRD and had a follow-up period of at least 6 months at the Keimyung University Dongsan Medical Center between January 2017 and December 2018. This study was performed in accordance with the Declaration of Helsinki and all research was performed in accordance with relevant guidelines/regulations. This study was approved by the Keimyung University Hospital Institutional Review Board (IRB no. 2020-03-037). Informed consent was obtained from all individual patients included in this study. Patients were excluded if they had an uninterpretable OCT, a history of vitrectomy or buckling surgery, or ocular diseases affecting visual acuity and visual field. With swept source OCT (Topcon DRI OCT Triton, SS-OCT, Tokyo, Japan), central macular thickness (CMT), the height of subretinal fluid (SRF), presence or absence of undulation, and ellipsoid zone (EZ) integrity were measured in the preoperative state. CMT was defined as the distance between the umbo and the outer border of the retinal pigment epithelium (RPE). The peak height of SRF was measured between the inner border of the RPE and the highest outer border of the photoreceptor outer segment. These distances were measured perpendicular to the RPE by a ruler in the IMAGEnet 6 (version 1.25; Topcon, Tokyo, Japan) software. Central retinal thickness (CRT) was calculated by substracting the SRF height from CMT. Disruption of the EZ integrity was judged based on the discontinuity of the EZ in OCT. ORU was defined as a change in the outer retinal gradient (negative to positive) of ≥ 3 within a diameter of 6 mm (Fig. 1). Symptom duration was defined as the time between the onset of subjective visual field loss and time of retinal detachment detection on examination. The patients were classified into acute (< 10 days), subacute (10–30 days) and chronic (> 30 days) groups, according to symptom duration.

BCVA was measured preoperatively and 1, 3, and 6 months postoperatively by the Snellen chart. BCVA was then converted to the logarithm of the minimum angle of resolution (logMAR).

For subgroup analysis in the macula-off RRD group, preoperative OCT findings and postoperative clinical data for 30 eyes that had pars plana vitrectomy were included. Eyes with intravitreal silicone oil tamponade during surgery, and with postoperative complications such as proliferative vitreoretinopathy (≥ grade C), re-detachment of the retina, or endophthalmitis, were excluded.

All statistical analyses were performed with the SPSS software (version 25; IBM Corp., Armonk, NY). The correlation of undulation and retinal detachment duration was determined with the Chi-square test and Fisher's exact test. The relationship between preoperative OCT findings and postoperative BCVA was determined with Pearson's product moment correlation coefficient, Student’s t-test and the Kruskal–Wallis test.

### Conference presentation

This study was partially presented as a poster at the 122nd Annual Meeting of the Korean Ophthalmological Society 2019.

## Results

One hundred and fourteen patients were enrolled. Sixty-two patients were male (54.4%) and 52 were female (45.6%). The mean (± standard deviation (SD)) age was 53.79 ± 13.48 years. The mean (± SD) symptom duration was 12.09 ± 14.25 days. According to symptom duration, groups were classified as acute (n = 65, 57.0%), subacute (n = 38, 33.3%), and chronic (n = 11, 9.7%). There were 49 patients with macula-off RRD (43.0%) and 65 patients with macula-on RRD patients (57%). On preoperative OCT, ORU was detected in 56 eyes (49.1%) (Table 1).

**Table 1:** Patient characteristics and comparison of the presence or absence of outer retinal undulation with all RRD.

Baseline data		Outer retinal undulation		*P*-value
		Absence (n = 58)	Presence (n = 56)	
Age, mean ± SD, years		56.10 ± 12.82	51.39 ± 13.83	0.062^1^
Sex, eyes, n (%)	Male	29 (46.8)	33 (53.2)	0.354^2^
	Female	29 (55.8)	23 (44.2)	
Eyes, eyes, n (%)	OD	26 (53.1)	23 (46.9)	0.709^2^
	OS	32 (49.2)	33 (50.8)	
Symptom duration, ± SD, days		11.59 ± 15.52	12.61 ± 12.92	0.704^1^
Symptom duration, eyes, n (%)	Acute (< 10 days)	40 (61.5)	25 (38.5)	0.001^2^
	Subacute (10–30 days)	10 (26.3)	28 (73.7)	
	Chronic (> 30 days)	8 (72.7)	3 (27.3)	
Macula-off, eyes, n (%)	On	37 (56.9)	28 (43.1)	0.185^2^
	Off	21 (42.9)	28 (53.1)	
Retinal hole size, ± SD, disc diameter		1.35 ± 1.39	1.28 ± 1.21	0.779^1^
Pre-op BCVA (LogMAR) ± SD		0.75 ± 0.77	1.08 ± 1.12	0.070^1^
Post-op 6 months BCVA (LogMAR) ± SD		0.24 ± 0.25	0.25 ± 0.21	0.750^1^
Surgical procedure, eyes, n (%)	PPV	30 (43.5)	39 (56.5)	0.118^3^
	Buckling	26 (61.9)	16 (38.1)	
	PPV + buckling	2 (66.7)	1 (33.3)	

^1^Student’s t-test.

^2^Chi-square test.

^3^Fisher's exact test.

*RRD* rhegmatogenous retinal detachment, *OD* oculus dexter, *OS* oculus sinister, *BCVA* best-corrected visual acuity, *PPV* pars plana vitrectomy.

**Figure 2:**
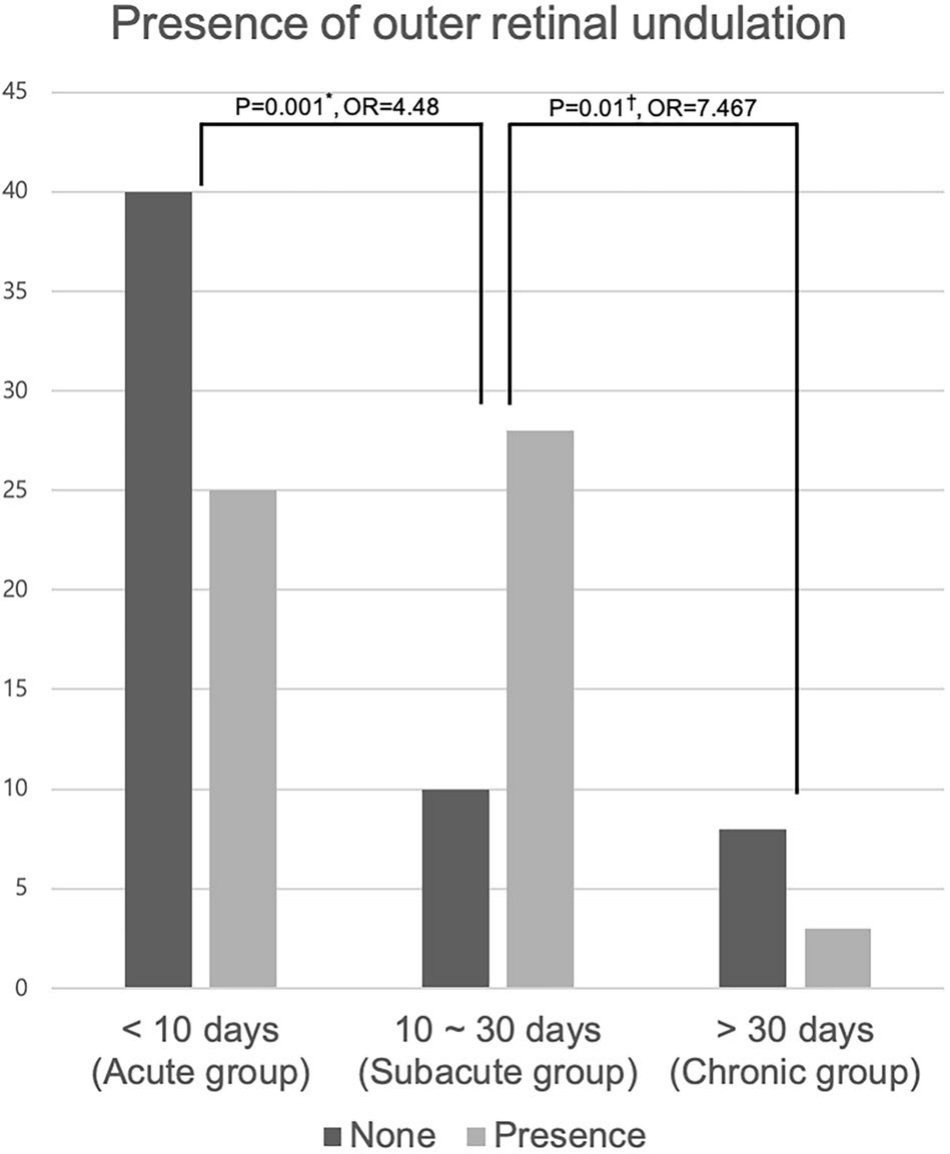
Presence of outer retinal undulation (ORU) in each group on preoperative optical coherence tomography. *Chi-square test; ^†^Fisher's exact test.

In the univariate analysis, age, sex, macula status, retinal break size, preoperative BCVA, and postoperative BCVA were not significantly different with respect to on the presence or absence of ORU. ORU was however significantly associated with symptom duration (P = 0.001). The subacute group had a higher incidence of ORU (73.7%) than the acute (P = 0.001, OR = 4.48) and chronic (P = 0.01, OR = 7.467) groups. There was no significant difference between the acute and chronic groups in the incidence of ORU (Fig. 2) (Table 1). In the multivariate analysis, additionally, the absence of ORU was significantly associated with older age and better preoperative BCVA (Table 2). Age, sex, and retinal hole size were not significantly different with respect to symptom duration. The longer the symptom duration, the higher the preoperative BCVA and the higher the macula-off rate (Table 3).

**Table 2:** Multivariate analysis of possible factors associated with outer retinal undulation with all RRD.

		Adjusted OR	95% CI	*P* value
Age		0.965	0.934–0.997	0.025*
Sex Eyes				0.193
				0.231
Symptom duration				0.066
Symptom duration	Acute (< 10 days)	0.078	0.014–0.442	0.002*
	Subacute (10–30 days)	1		
	Chronic (> 30 days)	0.320	0.125–0.442	
Macula-off				0.629
Retinal hole size				0.817
Pre-op BCVA		1.752	1.025–2.995	0.030*
Post-op 6 months BCVA				0.963
Surgical procedure				0.487

*OR* odds ratio, *CI* confidence interval, *BCVA* best-corrected visual acuity, *RRD* rhegmatogenous retinal detachment.

*Statistically significant as calculated using binary logistic regression analysis with backward elimination (Nagelkerke R^2^ = 0.236, *P* = 0.259 by the Hosmer–Lemeshow test for goodness of fit).

**Table 3:** Patient characteristics and the comparison of symptom duration groups with all RRD.

Baseline data		Symptom duration			*P*-value
		Acute (< 10 days)	Subacute (10–30 days)	Chronic(> 30 days)	
Age, mean ± SD, years		54.58 ± 12.28	52.05 ± 13.51	53.55 ± 19.83	0.600^1^
Sex, eyes, n (%)	Male	38 (58.5)	19 (50.0)	5 (45.5)	0.589^2^
	Female	27 (51.5)	19 (50.0)	6 (55.5)	
Eyes, eyes, n (%)	OD	27 (51.5)	18 (47.4)	4 (36.4)	0.814^2^
	OS	38 (58.5)	20 (52.6)	7 (63.6)	
Macula-off, eyes, n (%)	On	45 (69.2)	18 (47.4)	2 (18.2)	0.002^3^
	Off	20 (40.8)	20 (52.6)	9 (71.8)	
Retinal hole size, ± SD, disc diameter		1.44 ± 1.36	1.26 ± 1.34	0.81 ± 0.34	0.315^1^
Pre-op BCVA (LogMAR) ± SD		0.58 ± 0.68	1.20 ± 1.13	1.85 ± 0.96	0.000^1^

^1^One-way ANOVA test.

^2^Chi-square test.

^3^Fisher's exact test.

*RRD* rhegmatogenous retinal detachment, *OD* oculus dexter, *OS* oculus sinister, *BCVA* best-corrected visual acuity.

In the subgroup analysis of macula-off RRD with pars plana vitrectomy (n = 30), the mean ± SD age was 57.80 ± 9.94 years and the mean ± SD symptom duration was 12.77 ± 12.41 days. On preoperative OCT, ORU was detected in 20 of these eyes (66.7%). Twelve patients (40.0%) from this subgroup had disruption of the EZ integrity, and 18 patients (60.0%) had normal EZ integrity. The mean ± SD CRT was 0.26 ± 0.15 mm, and the mean ± SD baseline SRF height was 1.35 ± 0.91 mm (Table 4).

**Table 4:** Characteristics of patients with macula-off RRD with PPV and preoperative factors influencing BCVA 6 months postoperatively.

Baseline data			Post-operation BCVA (LogMAR) ± SD	*P*-value
Age, mean ± SD, years		57.80 ± 9.94	0.31 ± 0.24	0.397^1^
Sex, eyes, n (%)	Male	18 (60.0)	0.32 ± 0.21	0.583^2^
	Female	12 (40.0)	0.30 ± 0.29	
Laterality, eyes, n (%)	OD	14 (46.7)	0.35 ± 0.25	0.313^2^
	OS	16 (53.3)	0.28 ± 0.24	
Lens status, eyes, n (%)	Phakic	18 (60.0)	0.27 ± 0.24	0.295^2^
	Pseudophakic	12 (40.0)	0.38 ± 0.23	
Tamponade gas, eyes, n (%)	None	4 (13.3)	0.27 ± 0.21	0.152^3^
	SF_6_	21 (70.0)	0.36 ± 0.26	
	C3F8	5 (16.7)	0.16 ± 0.09	
Symptom duration, ± SD, days		12.77 ± 12.41	0.31 ± 0.24	0.584^1^
Extent of RRD, eyes, n (%)	< 1/4	0 (0.0)		0.149^3^
	1/4–1/2	12 (40.0)	0.25 ± 0.23	
	1/2–3/4	14 (46.7)	0.31 ± 0.24	
	≥ 3/4	4 (13.3)	0.50 ± 0.22	
Preop BCVA (LogMAR) ± SD		1.89 ± 0.89	0.31 ± 0.24	0.376^4^
Central retinal thickness, mm		0.26 ± 0.15	0.31 ± 0.24	0.284^1^
Baseline SRF, mm		1.35 ± 0.91	0.31 ± 0.24	0.432^1^
EZ integrity, eyes, n (%)	Disruption	12(40.0)	0.45 ± 0.25	0.014^2^
	Normal	18 (60.0)	0.22 ± 0.19	
Outer retinal undulation, eyes, n (%)		20 (66.7)	0.31 ± 0.24	0.920^2^
Post-op 1 month BCVA (LogMAR) ± SD		0.67 ± 0.39	0.31 ± 0.24	
Post-op 3 months BCVA (LogMAR) ± SD		0.40 ± 0.25	0.31 ± 0.24	

*RRD* rhegmatogenous retinal detachment, *PPV* pars plana vitrectomy, *OD* right eye, *OS* left eye, *BCVA* best-corrected visual acuity, *SRF* subretinal fluid, *EZ* ellipsoid zone, *ILM* inner limiting membrane.

^1^Pearson's product moment correlation coefficient.

^2^Student`s t-test.

^3^Kruskal-Wallis test.

^4^Paired sample t-test.

Only EZ integrity was significantly associated with postoperative BCVA (P = 0.014). Symptom duration, extent of detachment, ORU, CRT, and SRF had no significant correlation with postoperative BCVA (Table 4).

## Discussion

In previous studies, many preoperative factors have been reported to be significantly correlated with visual outcome for patients with RRD. These factors include patient age, preoperative visual acuity, duration of macular detachment, extent of retinal detachment, height of macular detachment, location and size of the retinal break, and proliferative vitreoretinopathy^1–3,7^. After the advent of OCT, the integrity of the photoreceptor outer segment layer, external limiting membrane integrity, and height of retinal detachment at the central fovea were reported to be correlated with postoperative BCVA^8–11^. In our study, EZ integrity was significantly associated with postoperative BCVA in patients with macula-off RRD with pars plana vitrectomy (P = 0.014) (Table 4).

ORU is another OCT finding in patients with RRD^10,12,14^. Cho et al. found that the presence of ORU on preoperative OCT is predictive of poorer visual acuity both preoperatively and one month postoperatively^14^. However, Kang et al., Hagimura et al., and Karacorlu et al. could not find a correlation between the presence of ORU on preoperative OCT and postoperative visual outcome^10,12,15^. A study of experimental RRD in the owl monkey, the authors reported that edema could cause posterior protrusion and a wavy, shagreen-like appearance^16^. ORU is presumed to come from the disparity of the amount of edema between the inner and outer retina in RRD. The retinal edema seen in RRD develops from subretinal fluid and retinal damage following retinal detachment. The disparity of edema is probably because retinal damage is more severe in the outer retina than the inner retina due to the physiologic dependency of the outer retina on the RPE and choroid; SRF is adjacent to the outer retina, and tangential expansion of the inner retina is possibly limited by the internal limiting membrane.

Many studies have reported that poorer visual outcomes are associated with a longer duration of preoperative retinal detachment. Hassan et al. did not find an effect on postoperative visual acuity in the first 10 days of detachment, but did find a decrease in visual outcome 10 days after symptoms were reported^2^. Enders P et al. reported poorer visual acuity after symptoms which lasted more than 30 days compared to a shorter symptom duration^3^. Other studies have defined days 5 or 10 after the onset of symptoms as important thresholds for adverse visual outcomes^4–7^. The current study found that the subacute group (10–30 days) had a higher incidence of ORU than did the other groups. Based on our results, ORU can be used to determine retinal detachment duration in patients with unknown duration of symptoms.

In the current study, ORU did not have a significant influence on visual outcome in patients with macula-off RRD. As mentioned above, in previous studies, there has been controversy about the relationship between ORU and visual outcome. This may be due to the different compositions of the enrolled patients in each study. For example, if many acute cases are included, ORU could be a prognostic indicator of bad vision. However, if many chronic cases are included, ORU could be a good prognostic factor for vision, or may have no significance if the distribution of case duration is even. In their study, Cho et al. included only acute and subacute cases (average of 11 days, range 1–30 days) and found that the presence of ORU on preoperative OCT was predictive of poorer visual acuity^14^. However, Kang et al. (average 12.2, range 0–60 days) and Hagimura et al. (average 16 days, range 2–60 days) did not find a correlation between ORU and postoperative visual outcome^10,12^. In their study, Karacorlu M et al. primarily included only acute cases (average 6 days, range 3–13 days) and did not find a correlation between the presence of ORU and postoperative BCVA^15^.

In the multivariate analysis, the presence of ORU was significantly associated with younger age and better preoperative BCVA. Due to the more rapid progression to atrophy in older patients, the group with ORU is thought to be younger and have better BCVA. The number of patients in the chronic group was relatively small; the absence of ORU had a better preoperative BCVA associated with the acute group (Table 2).

The main limitations of this study are as follows: the short follow-up duration of 6 months; the retrospective study design; the fact that patients with severe bullous RRD were excluded from our study, because OCT was unable to image their eye; our inability to compare the states of the lenses or the surgical methods under the same conditions, due to the small sample size of eyes with macula-off RRD (n = 30); and the cross-sectional nature of this study, which limited our ability to fully explain the time course of ORU. Further longitudinal studies are needed to clarify the relationship between morbidity and ORU.

In conclusion, the presence of ORU on preoperative OCT may be associated with the duration of retinal detachment. Integrity of the EZ is useful to predict postoperative BCVA in macula-off RRD, and preoperative OCT images are a useful tool for the assessment of the duration and prognosis of RRD.

## Data Availability

The datasets generated during and/or analyzed during the current study are available from the corresponding author on reasonable request.
